# A novel polypeptide encoded by the circular RNA ZKSCAN1 suppresses HCC via degradation of mTOR

**DOI:** 10.1186/s12943-023-01719-9

**Published:** 2023-01-23

**Authors:** Runjie Song, Shuoqian Ma, Jiajia Xu, Xin Ren, Peilan Guo, Huijiao Liu, Peng Li, Fan Yin, Mei Liu, Qiang Wang, Lei Yu, Jiali Liu, Binwei Duan, Nafis A. Rahman, Sławomir Wołczyński, Guangming Li, Xiangdong Li

**Affiliations:** 1 State Key Laboratory of Agrobiotechnology, College of Biological Sciences, China Agricultural University, Beijing, 100193 China; 2 Department of Oncology, The Second Medical Centre & National Clinical Research Center of Geriatric Disease, Chinese PLA General Hospital, Beijing, 100071 China; 3 Department of Pathology, Chinese PLA General Hospital, Beijing, 100071 China; 4 Department of Urology, Peking University People’s Hospital, Beijing, 100044 China; 5 Department of Thoracic Surgery, Beijing Tongren Hospital, Capital Medical University, Beijing, 100730 China; 6 Department of General Surgery CenterBeijing You An Hospital, Clinical Center for Liver Cancer, Capital Medical University, Beijing, China; 7 Department of Physiology, Institute of Biomedicine, University of Turku, Turku, Finland; 8 Department of Reproduction and Gynecological Endocrinology, Medical University of Bialystok, Bialystok, Poland

**Keywords:** HCC, circZKSaa, Sorafenib, mTOR, Biomarker

## Abstract

**Background:**

hsa_circ_0001727 (circZKSCAN1) has been reported to be a tumor-associated circRNA by sponging microRNAs. Intriguingly, we found that circZKSCAN1 encoded a secretory peptide (circZKSaa) in the liver. The present study aims to elucidate the potential role and molecular mechanism of circZKSaa in the regulation of hepatocellular carcinoma (HCC) progression.

**Methods:**

The circRNA profiling datasets (RNA-seq data GSE143233 and GSE140202) were reanalyzed and circZKSCAN1 was selected for further study. Mass spectrometry, polysome fractionation assay, dual-luciferase reporter, and a series of experiments showed that circZKSCAN1 encodes circZKSaa. Cell proliferation, apoptosis, and tumorigenesis in nude mice were examined to investigate the functions of circZKSaa. Mechanistically, the relationship between the circZKSaa and mTOR in HCC was verified by immunoprecipitation analyses, mass spectrometry, and immunofluorescence staining analyses.

**Results:**

Receiver operating characteristic (ROC) analysis demonstrated that the secretory peptide circZKSaa encoded by circZKSCAN1 might be the potential biomarker for HCC tissues. Through a series of experiments, we found that circZKSaa inhibited HCC progression and sensitize HCC cells to sorafenib. Mechanistically, we found that the sponge function of circZKSCAN1 to microRNA is weak in HCC, while overexpression of circZKSaa promoted the interaction of FBXW7 with the mammalian target of rapamycin (mTOR) to promote the ubiquitination of mTOR, thereby inhibiting the PI3K/AKT/mTOR pathway. Furthermore, we found that the high expression of cicZKSCAN1 in sorafenib-treated HCC cells was regulated by *QKI-5*.

**Conclusions:**

These results reveal that a novel circZKSCAN1-encoded peptide acts as a tumor suppressor on PI3K/AKT/mTOR pathway, and sensitizes HCC cells to sorafenib via ubiquitination of mTOR. These findings demonstrated that circZKSaa has the potential to serve as a therapeutic target and biomarker for HCC treatment.

**Supplementary Information:**

The online version contains supplementary material available at 10.1186/s12943-023-01719-9.

## Background

The main histological type of liver cancer, hepatocellular carcinoma (HCC), is the cause of 75–85% of primary liver cancer cases and has ranked second in terms of cancer mortality in humans [1]. Most HCC patients are first diagnosed at an advanced stage, and thereby the survival rate is poor [2, 3]. Meanwhile, the treatments for HCC are limited. Sorafenib is the sole first-line therapeutic drug in the treatment of HCC for 10 years, as it can improve the survival rate of patients with advanced HCC [4]. During the past few years, surgical approaches and systematic drug treatments have been developed. For example, anti-angiogenesis drugs such as sunitinib and brivanib have been approved as complements to sorafenib [5, 6]. However, the overall survival of HCC patients remains poor, and the early diagnosis of HCC has always been a major challenge [2]. Therefore, the developments of early diagnostic markers and mechanism-based therapies are critical and needed to be further studied.

Circular RNAs (circRNAs) generated by back-splicing of precursor-mRNAs are a type of molecules of covalently closed loop structure lacking 5′-3′ polarity and polyadenylated tail. Although the function of circRNAs has not been fully understood, recent studies showed that circRNAs executed various functions such as acting as protein or miRNA sponges, encoding polypeptides, or forming a stable RNA-protein complex to regulate the downstream biological processes [7]. Many circRNAs also play very important regulatory functions in HCC. For example, has_circ_104348 and circARNT2 can act as a sponge in HCC cells to adsorb microRNA and affect the proliferation and migration of HCC cells [8, 9]. circDLC1 and circBACH1 can inhibit the deterioration of HCC by binding to ELAV-like protein 1 [10, 11]. However, the understandings of the roles of circRNAs in HCC studies are only at the preliminary level, a large knowledge gap needs to be further explored.

circZKSCAN1 has recently been shown to act as a microRNA sponge in various studies. For example, circZKSCAN1 up-regulated cyclin-dependent kinase inhibitor 1A expression by sponging miR-1178-3p, which suppressed the aggressive biological behaviors in bladder cancer [12]. circ-ZKSCAN1/miR-330-5p/FAM83A feedback loop promoted the progress of NSCLC [13]. It has been reported that circZKSCAN1 inhibited the growth, migration, and invasion of HCC [14]. Zhu et al. found that circZKSCAN1 suppressed the transcriptional activity of the Wnt/β-catenin signal pathway through competitively binding to fragile X mental retardation protein (FMRP) in HCC [12]. In contrast, we found that circZKSCAN1 might not sponge microRNA in HCC. Intriguingly, we demonstrated that circZKSCAN1 could be translated into a novel peptide (circZKSaa), which has a potential diagnostic value for HCC, and this peptide could sensitize HCC cells to sorafenib treatment. circZKSaa facilitated mTOR ubiquitination through interaction with FBXW7, and eventually suppressed HCC cell proliferation. Our results may not only expand the better understanding of circZKSAN1 in HCC but also indicate that circZKSaa might be a new diagnostic and therapeutic target for the treatment of HCC.

## Materials and methods

### Patients and tissue samples

HCC and normal tissues of patients were used as described in our previous study [15]. Both tissues were frozen in liquid nitrogen and extracted by RIPA Lysis Buffer (GenStar, China). All patients underwent surgery in the Chinese PLA General Hospital between 2018 and 2020 and did not receive chemotherapy or radiotherapy before surgery. The study was approved by the Ethical Review Committee of Chinese PLA General Hospital. All the patients had signed the informed consent.

### Related software usage and information collation

The method of sorting data was performed according to our previous study [15]. Briefly, the expression profiling data were downloaded from the NCBI public database GSE143233, GSE140202 and GSE114564. The circRNAs were discovered by Linux’s CIRIquant, find_circ and other softwares and then used CIRIquant to quantify circRNA expression.

### Cell culture, stably transfected cells selection

The human HCC cell lines HCC-LM3, SNU-398, Hep3B, Huh-7, SNU-387, human embryonic kidney cells (293 T) and human normal liver cell line THL-2 were purchased from National Infrastructure of Cell Line Resource and maintained in Dulbecco’s modified Eagle’s medium (Invitrogen, USA) supplemented with 10% fetal bovine serum (Gibco, USA) and L-glutamine (Gibco, USA) at 37 °C with 5% CO_2_. Lentiviral vectors (Addgene, USA) were co-transfected with packaging plasmids psPAX2 and pMD2.G (Addgene, USA) into 293 T cells. Infectious supernatant was harvested at 48 h and 72 h after transfection and filtered through 0.45 μm filters (Millipore, USA). Cells were infected by recombinant lentivirus for 48 h and then selected by the ppropriate concentration of puromycin (Aladdin, China) for 72 h.

### Vectors construction, stable transfection, and dual-luciferase reporter gene assay

The whole sequence of circZKSCAN1 and mutated circZKSCAN1 start codon was cloned into PL05-ciR (GENESEED, China) vector. Specific shRNAs for circZKSCAN1 designed to target the covalently closed junction was amplified into PLKO.1-TRC plasmid (Tsingke, China). The whole sequence of circZKSaa and circZKSaa with mutated circZKSaa start codon was amplified into pLV-(2A)-puro vector (Tsingke, China). The whole sequence of mTOR, FBXW7 and mutated mTOR was amplified into pCDN3.1(+) or pCMV-HA vector (Tsingke, China). The two potential IRES sequences of circZKSaa were cloned into Psi-check2 vector (Promega, USA). Both plamids were transfected using LIPO 3.0 (HANBIO, China) at a final concentration of 20 nM. Transfected cells were harvested for indicated analyses 2 d after transfection. Dual luciferase reporter assay used Hela cells, luminescent reaction solution (Galen, China), multi-function microplate reader (Tecan, China). The firefly luciferase readings were measured and the corrected relative luciferase activity was obtained by dividing the desired fluorescence value according to the vector characteristics.

### Western blot

Cells extracts were prepared and analyzed as previously described [16]. Western blotting was performed by anti-ZKSCAN1 (Bioss, P17029), FBXW7 Rabbit Polyclonal Antibody (ABclonal, A5872), mTOR Mouse Monoclonal antibody (Proteintech, 66,888–1-lg), β-Actin Rabbit mAb (ABclonal, AC026), anti-His Mouse Monoclonal antibody (ABclonal, AE003), HA-tag antibody (ABclonal, AE008), anti DDDDK-Tag Mouse Monoclonal antibody (ABclonal, AE005), anti-GAPDH (Sangon Biotech, D110016), AKT Polyclonal Antibody (Proteintech, 51,077–1-AP), Phospho-Akt (Ser473) (Cell Signaling Technology, 4060), Phospho-Akt (Thr308) (Cell Signaling Technology, 13,038), Phospho-p44/42 MAPK (Erk1/2) (Thr202/Tyr204) (Cell Signaling Technology, 4370), ERK1/2 Monoclonal Antibody (67170–1-Ig), Phospho-mTOR (Ser2448) (Cell Signaling Technology, 5536), Phospho-PDK1 (Ser241) (Cell Signaling Technology, 3438), Phospho-MDM2 (Ser166) (Cell Signaling Technology, 3521), Phospho-p70 (S6) Kinase (Thr389) (Cell Signaling Technology, 97,596), MDM2 (Proteintech, 66,511–1-Ig), PDK1 (Proteintech, 18,262–1-AP), P70 (S6K) (Proteintech, 14,485–1-AP), Biotinylated Rabbit anti-Goat IgG (ZSGB-BIO, ZB-2050) and Biotinylated Mouse anti-Goat IgG (ZSGB-BIO, ZB-2020) antibodies. The antigen-antibody complexes were visualized by chemiluminescence.

### Coimmunoprecipitation (Co-IP)

The cells were lysed by IP-lysis buffer (50 mM Tris (pH 7.4), 150 mM NaCl, 0.1 mM EDTA, 20% glycerol, 0.2% NP-40, 0.1% SDS, protease and phosphatase inhibitors). After freezing and high-speed centrifugation to remove cell debris, the supernatant of cell lysates was incubated with indicated antibodies in 4 °C overnight. Then, the pre-cleared protein A/G beads (GE Healthcare, USA) were incubated with the supernatant for 3 h at room-tempreture. The collected proteins were analysis by Western blot.

### Immunofluorescence assay

The detailed experimental methods can be found in our previously published articles [17]. Briefly, the cells were fixed with 4% paraformaldehyde for 30 min at room temperature and then treated with 0.2% Trition X-100 for 30 min. Cells were incubated with 10% goat serum for 60 min at room temperature and incubated with the HA and His antibodies overnight at 4 °C. FITC-conjugated anti-rabbit antibody and TRITC conjugated anti-mouse antibody were incubated with cells at room temperature for 60 min. Then, cells were stained with DAPI for 30 min at room temperature. Finally, the fluorescence confocal microscope was used to observe and photograph.

### RNA immunoprecipitation (RIP)

RIP assays were performed with using a Magna RIP Kit (Millipore, USA) following the manufacturer’s instructions. In brief, 2 × 10^7^ 293 T cells were lysed in 100 μL RIP lysis buffer containing a proteinase inhibitor cocktail and RNase inhibitor (Promega, USA). Lysate was treated with DNase I (Roche Life Science, Switzerland) at 37 °C for 10 min and centrifuged at 12,000×g for 30 min. The lysate was then diluted with 900 μl RIP buffer and incubated for 3 h with 5 μg anti-Ago2 (Abcam, ab57113, USA) antibody that was pre-bound on magnetic beads. After treated with proteinase K at 37 °C for 30 min, the immunoprecipitated RNA was extracted using TRIzol® regent (Invitrogen, Life Technologies Inc., Germany) according to the manufacturer’s instructions.

### Cell proliferation assays

Firstly, HCC-LM3 and Hep3B cells were seeded into 96-well plates. The cells were collected and used Cell Counting Kits-8 (Beyotime, China) according to the manufacturer’s protocol. The absorbance was measured at OD450. Secondly, HCC cells were collected and fixing with ice cold acetone. After washing and blocked with BSA buffer (1% BSA, 0.3% Triton, 1 × PBS (Solarbio, China), the cells were incubated with KI67 (1:1000, CST, USA) antibody at 4 °C overnight. After washing with PBS, cells were incubated with corresponding fluorescent secondary antibody, and counterstained for nuclei using 4′, 6-diamidino-2-phenylindole (DAPI) (Solarbio, China). All experiments were performed in triplicate.

### Immunofluorescence assay and TdT-mediated dUTP Nick-end labeling (TUNEL) staining

For immunofluorescence, the cells were fixed with 4% paraformaldehyde for 10 min, and permeabilized with 0.1% Triton X-100 for 15 min at room temperature, and then blocked with PBS containing 5% bovine serum albumin for 1 h at room temperature. The cells were incubated with certain antibody overnight at 4 °C. After rinsing with PBS, secondary antibody was applied for 1 h at 37 °C. The slices were counterstained with DAPI. For TUNEL staining, cells were fixed and transparented as described above. Then, the cell slices were operated according to the factory instructions of TUNEL staining kit (Beyotime, China).

### Polysome fractionation assay

The method for the isolation of HCC-LM3 was provided previously [15]. In short, the cells were treated with cycloheximide (CHX) for 5 min. Then polysome lysis buffer were applied to cells and incubated for 30 min. After centrifugation, the supernatant was loaded onto continuous 15–50% sucrose gradients buffer containing 50 U/ml RNase inhibitor (Solarbio, China). The cells fractions were collected using Brandel Fractionation System (USA). Extraction and total RNA of each fraction was measured by RT-PCR, and glyceraldehyde-3-phosphate dehydrogenase (GAPDH) served as a positive control.

### Mass spectrometry analysis

Proteins were separated by SDS-PAGE, the target bands were cut and digested with sequencing grade trypsin (Promega, USA). QExactive mass spectrometer (Thermo Fisher, USA) was used to analyze the digested peptides. The National Biotechnology Information Center’s non-redundant protein database and Mascot (Matrix Science, USA) were used to analyze peptide fragments.

### Flow cytometry analysis

Cells were trypsinizaed collected, and washed with ice-cold PBS. To detect the cell cycle, the cells need to be fixed with ethanol. Then RNaseA (Solarbio, China) was added, and followed incubation at 37 °C for 30 min. Propidium bromide (Beyotime, China) was then added, and cells were incubated at room temperature for 20 min. Finally, the sample analyzed by flow cytometry (BD, USA). To detect cell apoptosis, the cells were trypsinized and washed with ice-cold PBS, then stained with Cell Apoptosis Kit (Beyotime, China) according to manufacturer’s protocol. Flow cytometry (BD, USA) was used to analyze the cells samples.

### Murine and xenograft assay in vivo

Four-week-old female BALB/c nude mice were purchased from SIBEIFU Company (China) and maintained in accordance with the institutional policies. Mice were subcutaneously inoculated with HCC-LM3 (1 × 10^6^ cells per mouse; *n* = 5 or 6/group) stably expressed circZKSaa, circZKSCAN1-mut, vector, sh-scramble and sh-circZKSCAN1 plasmid. Tumor growth was measured after inoculated 25 d. All experimental operations were conducted in accordance with the Ethics Committee for Animal Experimentation of China Agricultural University. And Prevention and the permission number was SYXK (BEIING)-2019–0010.

### Drug treatment

For sorafenib, HCC-LM3 and Hep3B were reseeded in six-well plates (8 × 10^5^ cells per well) after transfected with vector for 24 h. Then cells were treated with 3 μM and 6 μM sorafenib (Sigma-Aldrich, USA), respectively. Cells were collected for RT-qPCR and Western blot analysis after 24 h infection. For CHX (Sigma-Aldrich, USA), before Western blot analysis, both HCC-LM3 and Hep3B were treated with 10 μM CHX for different duration (0 h, 2 h, 4 h, 6 h, 8 h).

### IC50 analysis

Several HCC transiently transfected cells were seeded in 96 wells (0.5 ~ 1 × 10^4^/well) and the experiments were repeated 3 times for each sample. The cells were incubated with gradient concentrations of sorafenib for 48–72 h. Then the Cell Counting Kit-8 (CCK-8) Kit (Beyotime, China) was used to assess cell viability.

### Ubiquitination assay

Both HCC-LM3 and Hep3B were transfected with HA-ubiquitin plasmid and with or without Flag-circZKSaa, Flag-FBXW7, sh-scramble and sh-circZKSCAN1, after transfected for 24 h, cells were treated with MG132 5 μg/mL (Sigma-Aldrich, USA) for 6 h. And cells were harvested for Co-IP with anti-Flag/mTOR-A/G beads.

### RNA and gDNA extraction, reverse transcription, mRNA quantification by reverse transcription quantitative polymerase chain reaction (qRT-qPCR) and Nucleo-cytoplasmic seperation

Genomic DNA (gDNA) was extracted from tissues and cultured cells using the TIANamp Genomic DNA Kit (TIANGEN, China) according to the manufacturer’s protocol. RNAs from the pulmonary tissue or cells after infection were extracted using the TRIzol® reagent (Cwbio, China) according to manufacturer’s instructions. cDNA was prepared from 1 μg RNA using 10 U of Moloney Murine Leukemia Virus (M-MLV) Reverse Transcriptase (Takara, Japan). RT-qPCR was performed on LightCycler480 (Roche Life Science, Switzerland) by using fast SYBR green master mix (GenStar, China) according to the manufacturer’s protocol. The expression levels of genes were normalized against GAPDH. The primer sets for these assays are described (Supplementary Table 1). RT-qPCR experiments were performed from three independent RNA preparations. The protein of nuclear and cytoplasmic was separated by PARIS Kit (Life technologies, USA) via the manufacturer’s protocol.

### Statistical analysis

The results were presented as the mean ± SEM (standard error of mean). All the statistical graphs were expressed with GraphPad Prism 8.0.3 (GraphPad Software Inc., USA). The results between the two groups were analyzed by two tailed paired or non-paired t-test, in which *P* values less than 0.05 means statistically significant.

## Result

### circZKSCAN1 is down-regulated in HCC tissues and is sensitive to sorafenib treatment

**Fig. 1:**
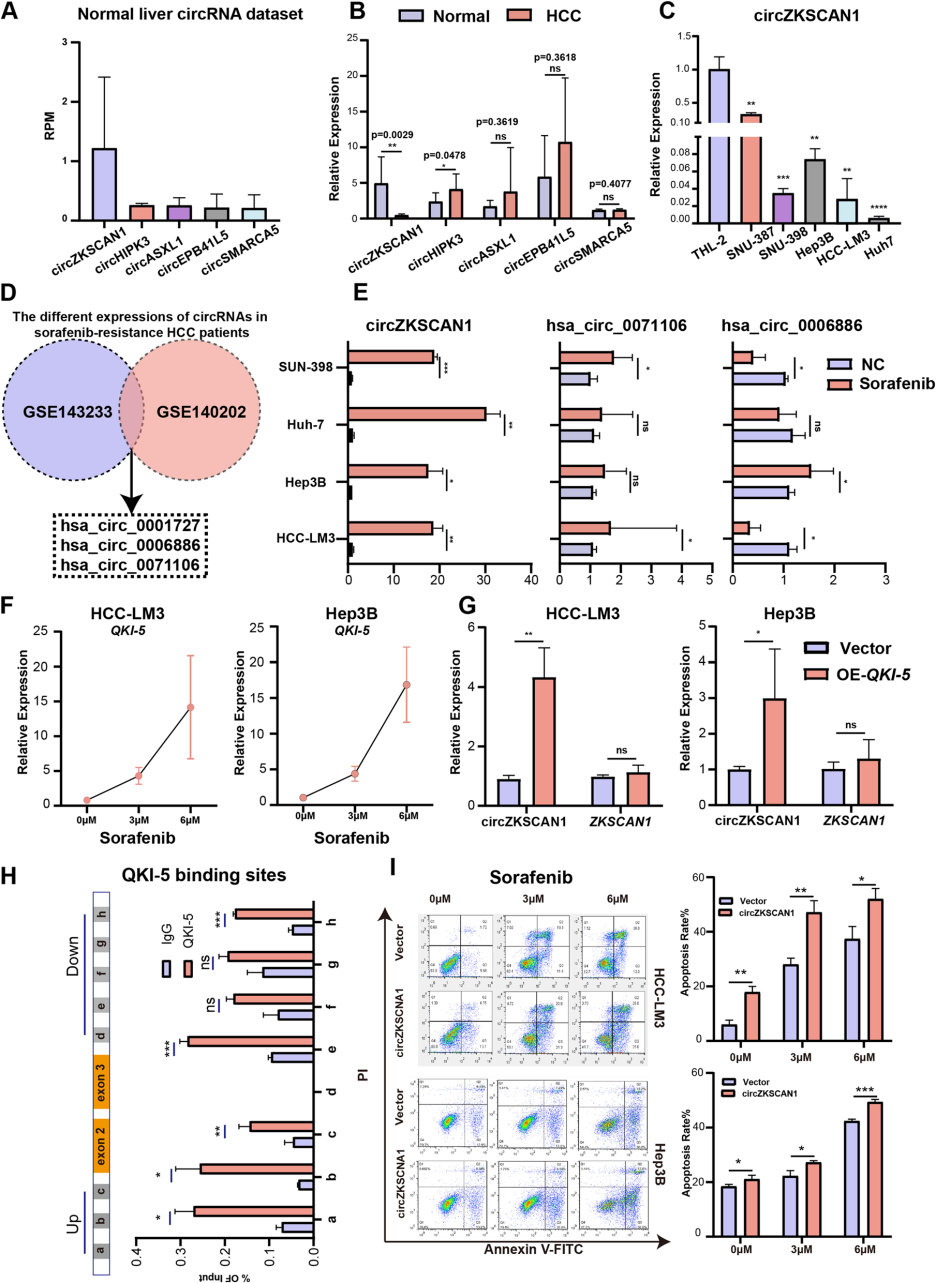
circZKSCAN1 is down-regulated in liver cancer tissues and is sensitive to sorafenib treatment. **A** Human adult normal liver datasets were analyses for differentially expressed circRNAs. The top 5 up-regulated circRNAs were at list. **B** Relative expression level of the top 5 up-regulated circRNAs in 30 pairs of HCC and normal tissues. **C** Relative expression level of the top 5 circRNAs in normal hepatocyte lines (THL-2) and HCC cell lines (SNU-387, SNU-398, HCC-LM3, Hep3B, Huh-7) by RT-qPCR analysis. **D** Schematic illustration exhibited the significantly different expressions circRNAs by overlapping GSE143233 and GSE140202. **E** The relative expression of circZKSCAN1, hsa_circ_0006886 and hsa_circ_0071106 in HCC cells with and without sorafenib treatment. **F** RT-qPCR analysis showed *QKI-5* expression level in HCC-LM3 and Hep3B after different concentrations of sorafenib treatment. **G** RT-qPCR analysis showed expression levels of circZKSCAN1 and ZKSCAN1 in overexpressed *QKI-5* HCC-LM3 and Hep3B cells. **H** RIP analysis showed expression levels of circZKSCAN1 in overexpressed *QKI-5* HCC-LM3 cells. **H** Analysis of apoptosis in HCC-LM3 and Hep3B with or without overexpressed circZKSCAN1 transfection by flow cytometry assays. T test was performed for significant analysis, significant differences are indicated with * for *p* < 0.05, ** for *p* < 0.01 and *** for *p* < 0.001

To identify the potential circRNAs differentially expressed between HCC and adjacent samples, we first reanalyzed the circRNA profiling dataset in the normal liver [18] and listed the top five circRNAs with the highest expression (Fig. 1A, Fig. S1A). Next, we detected the expressions of these 5 circRNAs by using 30 pairs of human HCC tissues (HCC and normal), and verified these expressions in both HCC cell lines (SNU-387, Hep3B, Huh-7, HCC-LM3 and SNU-398) and the normal liver cells line (THL-2) (Fig. 1B and C, Fig.S1B). We found that circZKSCAN1 was significantly down-regulated in HCC tissues and HCC cell lines (Fig. 1B and C).

As sorafenib is the first-line treatment and the sorafenib-resistance is the most common clinical problem for HCC [19]. We wanted to investigate whether circZKSCAN1 plays a role in sorafenib-resistant HCC patients, RNA-seq data (GSE143233 and GSE140202) of sorafenib-resistant HCC patient from GEO datasets (Gene Expression Omnibus, https://www.ncbi.nlm.nih.gov/geo/) have been analyzed by bioinformatics (Fig. 1D). The top three (hsa_circ_0001727, hsa_circ_0006886 and hsa_circ_0071106) differentially expressed circRNAs in all the two datasets were selected for the further study (Fig. 1E). Luckily, hsa_circ_0001727 is circZKSCAN1, which give us more confidence to dig out the molecular mechanism of circZKSCAN1 in the tumorigenesis of HCC. Futhermore, back splicing junction was validated by Sanger sequencing and the presence of circZKSCAN1, hsa_circ_0006886, and hsa_circ_0071106 were proved by RT-PCR (Fig. S1C and D).

To determine whether these circRNAs are functionally associated with sorafenib sensitivity in HCC cells, we examined their differential expression levels in a variety of HCC cells upon sorafenib treatment (3 μM). We detected that circZKSCAN1 was more highly expressed than rest of two circRNAs in all used HCC cell lines (Fig. 1E). These results draw our attention on figuring out the molecular action of circZKSCAN1 on the tumorigenesis of HCC. It has been reported that *QKI-5* plays a critical role in the formation of circZKSCAN1 [12]. Therefore, we speculate that sorafenib regulates the expression of circZKSCAN1 through *QKI-5*. We detected the expression level of *QKI-5* after treating HCC-LM3 and Hep3B cells with sorafenib, and found that the expression level of *QKI-5* was up-regulated (Fig. 1F). Futhermore, we overexpressed *QKI-5* to research the relation between *QKI-5* and circZKSCAN1. The results indicated that overexpressed *QKI-5* upregulated circZKSCAN1 (Fig. 1G). Indeed, bioinformatics predictions show that the flanking introns of ZKSCAN1unspliced precursor contained binding sites for *QKI-5*, which were validated by RIP and RT-qPCR. Our results demonstrated that *QKI-5* binds to the downstream and upstream of circZKSCAN1 (Fig. 1H). Therefore, we believe that sorafenib regulates the expression of circZKSCAN1 through upregulation of *QKI-5*.

By detecting both proliferation (IC50) and apoptosis (Flow cytometric assay) in two circZKSCAN1 overexpressed and knock down HCC cell lines with sorafenib treatment, we found that overexpressing circZKSCAN1 significantly decreased proliferation and promoted apoptosis of HCC (Fig. 1I, Fig. S1E), and knock down circZKSCAN1 significantly inhibited apoptosis of HCC cell lines under sorafenib treatment (Fig. S1F). All these results demonstrated that the circZKSCAN1 plays the important roles in HCC progression and treatment.

### circZKSCAN1 encodes a 206aa peptide

circZKSCAN1 executed various function such as protein/miRNA sponge to regulate downstream biological processes of cancers, especially in HCC [13, 20]. However, regulatory role of circZKSCAN1 in sorafenib sensitivity is unknown. Given that circRNAs have been shown to act as miRNA sponges in cancer cells, we sought to determine whether circZKSCAN1 can sponge microRNA in HCC. Through bioinformatics analysis with CircInteractome, we did not find the interaction possibility between circZKSCAN1 and AGO2 (Fig. S2A). Then, to verify whether circZKSCAN1 bind to AGO2 in in the cytosol of HCC cells, we observed no circZKSCAN1 enrichment in AGO2 precipitates by RIP experiments (Fig. S2B). Since miRNA-mediated gene silencing is carried out through AGO2 [21], we assumed that circZKSCAN1 might not work as microRNA sponge in HCC.

**Fig. 2:**
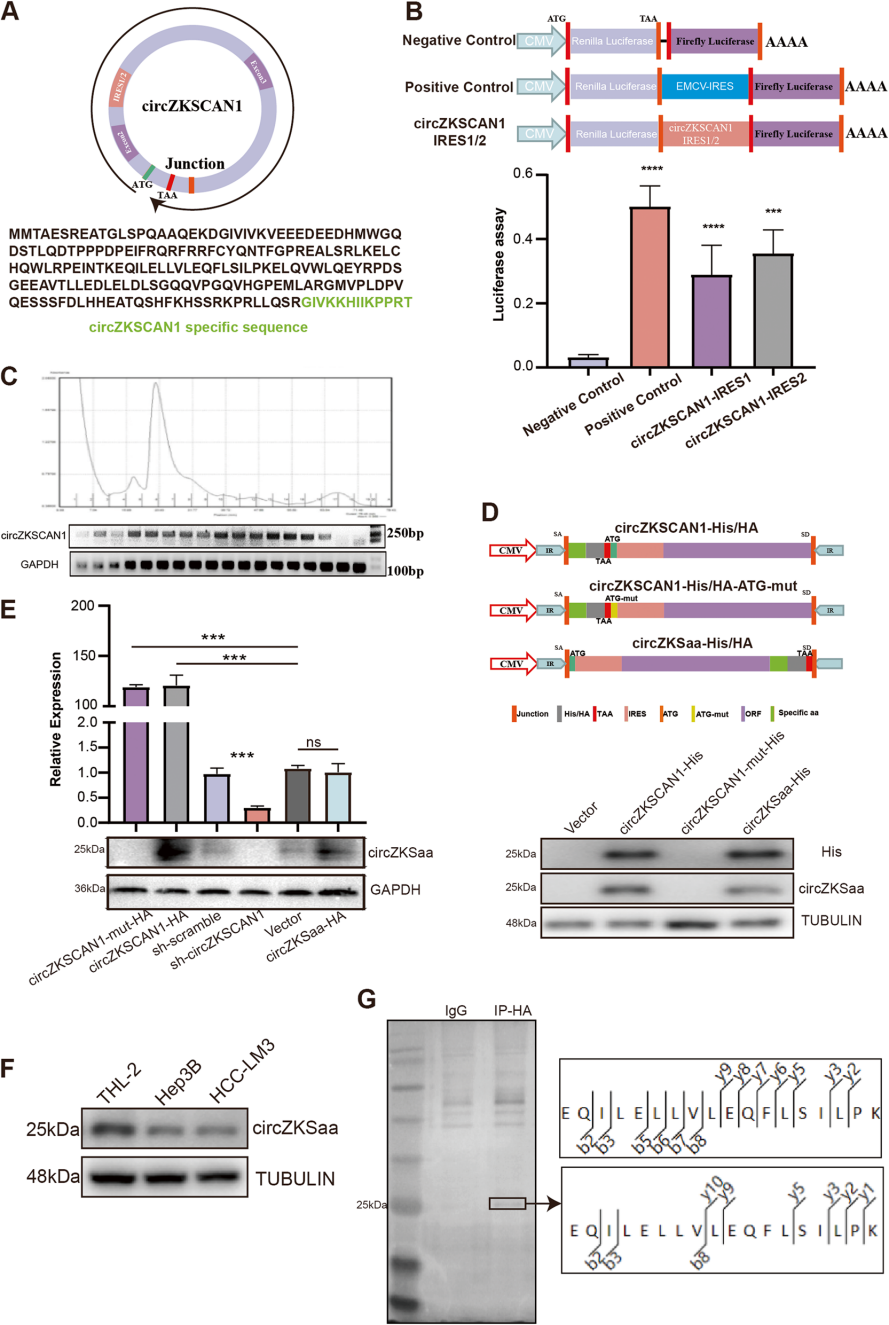
circZKSCAN1 encodes a 206aa peptides. **A** Vectors schematic diagram showed two IRSE sequences in circZKSCAN1 were cloned into Psi-check2 vector. **B** The relative luciferase activity of Luc/Rluc was used to tested circZKSCAN1 translation ability in HCC-LM3 cells, Encephalomyocarditis Virus (EMCV) IRES was used as a positive control, empty vector was served as a negative control. **C** Polysome fractionation and RT-PCR analysis after lysate of HCC-LM3 cells, GAPDH serve as a positive control. **D** Vectors schematic diagram showed that His/HA-labeled circZKSCAN1-mut, circZKSCAN1, Vector and circZKSaa plasmid. **E** Upper panel, RT-qPCR results of circZKSCAN1 on vector (circZKSCAN1-mut, circZKSCAN1, Vector and circZKSaa) transfected 293 T cells. Lower panel showed Western bolt assay of circZKSaa expression level on vector (circZKSCAN1-mut, circZKSCAN1, Vector and circZKSaa) transfected 293 T cells. **F** Western blot analyses showed circZKSaa expression of HCC-LM3, Hep3B and THL-2. **G** After Co-IP by HA antibody and separation by SDS-PAGE, the protein sample which stained by silver staining was analyzed by mass spectrometry. Data are presented as the mean ± SEM of three independent experiments

By using circRNADb (http://reprod.njmu.edu.cn/cgi-bin/circrnadb/circRNADb.php) database, we noticed that circZKSCAN1 has the internal ribosome entry site (IRES) and open reading frame (ORF) which may encode a 206 amino-acid polypeptide (Fig. S2C, Fig. 2A). To examine whether circZKSCAN1 is translatable, we used a tandem Renilla Luciferase-Luciferase (Rluc–Luc) reporter plasmid in which the Rluc ORF was driven by a cytomegalovirus promoter. We cloned two IRSE-site sequences (IRSE-1 and IRSE-2) between RLuc and Luc, and measured the luciferase activity of Luc relative to that of RLuc (Fig. 2B). The results showed that both IRES sequences exhibited significantly higher Luc/Rluc activities than the empty vector, which indicated that both IRSE-1 and IRSE-2 can induce ribosome entry (Fig. 2B). In order to analysis the distribution of this circZKSCAN1 translated peptide, we separated polysomes fractionation in the HCC-LM3 cell line. We found circZKSCAN1 was expressed in almost all fractions of HCC-LM3 polysomes (Fig. 2C).

To confirm the protein encoding capacity of circZKSCAN1, we constructed the circZKSCAN1 overexpressing plasmid named as plasmid circZKSCAN1. The linearized circZKSaa overexpressing plasmid was named as circZKSaa and its control plasmid as Vector. In addition, we also constructed a circZKSCAN1 knock-down plasmid sh-circZKSCAN1, a knockdown control plasmid sh-scramble and a plasmid circZKSCAN1-mut which overexpressed circZKSCAN1 with mutated start codon of circZKSaa (Fig. 2D). We found that 193 amino acids of circZKSaa were originated from ZKSCAN1, and the rest of 13 amino acids were unique (Fig. 2A). Therefore, we detected higher levels of circZKSaa expression in both overexpressed circZKSCAN1 and circZKSaa plasmid transfected 293 T cells, while no expression of circZKSaa was detected in the overexpressed circZKSCAN1-mut plasmid (Fig. 2D). Furthermore, we detected circZKSaa through Western blot by ZKSCAN1 antibody which has immunogen covering part of circZKSaa peptide (Fig. 2D). We found that the antibodies of HA/His and ZKSCAN1 recognized fragments of the same size in circZKSCAN1 and circZKSaa transfected cells (Fig. 2D and E). To further investigate whether this circZKSaa was encoded by circZKSCAN1, we tested the expression level of circZKSaa in normal liver and HCC cell lines. The results showed that THL-2 cells have higher endogenous circZKSaa compared to HCC cell lines (Fig. 2F). Moreover, using overexpressed circZKSCAN1 plasmid-transfected HCC-LM3 cells, we further confirmed that the peptide sequence of circZKSaa by mass spectrometry (Fig. 2G). In summary, these findings indicated that circZKSCAN1 did encode a 206aa protein in HCC cells.

### circZKSaa has the diagnostic value in HCC

**Fig. 3:**
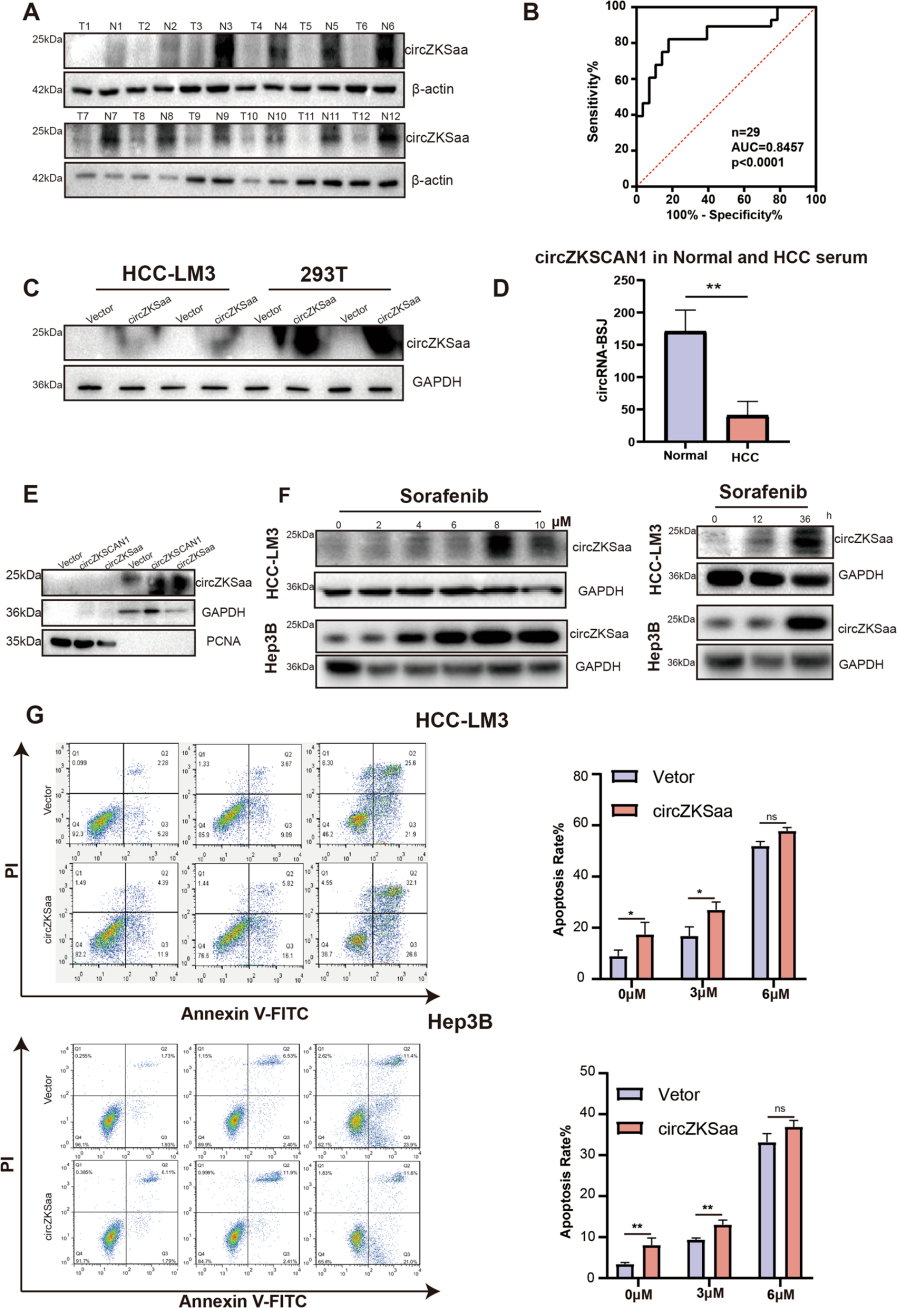
circZKSaa has diagnostic value in HCC. **A** The circZKSaa content was tested via Western blot in 12 pairs of HCC and normal tissues. β-actin was used to indicate the amount of loading proteins. **B** Diagnostic value of circZKSaa in 29 pairs of HCC and normal tissues by ROC curve. **C** After transfected circZKSaa plasmid in HCC-LM3 and 293 T cells, the circZKSaa expression level in cells supernatant. **D** The RNA-Seq databases of HCC patient’s serum databases was analyzed by Linux’s CIRIquant, find_circ and other softwares. **E** The location of circZKSaa was determined by Nucleo-cytoplasmic separation, GAPDH was used as the positive control of cytoplasmic protein, while PCNA was used as the positive control of nuclear protein. **F** Western blot assay showed circZKSaa expression level in HCC-LM3 cells after treated sorafenib with different concentrations and different duration. **G** Analysis of apoptosis in HCC-LM3 and Hep3B with or without circZKSaa transfection by flow cytometry assays. Quantitative data from three independent experiments are presented as mean ± SEM (error bars). T test was performed for significant analysis, significant differences are indicated with * for *p* < 0.05, ** for *p* < 0.01 and *** for *p* < 0.001

By examining the expression of circZKSaa in 12 paired HCC tumor tissues and normal tissues from patients, we found that the expression of circZKSaa was significantly decreased in tumor tissues compared to the paired normal tissues (Fig. 3A). ROC analysis was performed to determine the diagnostic value of circZKSaa in 29 pairs of HCC patient’s tissues (Fig. S2E), and the area under the ROC curve (AUC) showed that the specificity of diagnosis was 100% (Fig. 3B). Previous studies have shown that some circRNAs can be secreted into the serum, which could potentially serve as the biomarkers. Next, we showed that circZKSaa was secreted in the supernatant from circZSKaa transfected HCC-LM3 and 293 T cells (Fig. 3C). In addition, we found that the expression of circZKSCAN1 was low in the serum of HCC patients through database analysis (Fig. 3D). This suggests that circZKSCAN1 and/or the polypeptide circZKSaa could potentially serve as a biomarker for HCC patients. SDS-PAGE analysis of nuclear and cytoplasmic separation showed that circZKSaa was mainly expressed in the cytoplasm (Fig. 3E).

To figure out whether circZKSaa enhances the sensitivity of HCC cells upon sorafenib treatment, we pre-treated sorafenib in HCC-LM3 and Hep3B cell lines. The results demonstrated that the expression of circZKSaa was increased in sorafenib in both dose and time-dependent manners after 48 h treatment, which were consistent with the results from circZKSCAN1 overexpression assay (Fig. 3F). In order to detect whether circZKSaa would make HCC cells sensitive to sorafenib, we treated the circZKSaa-transfected HCC-LM3 and Hep3B cells with sorafenib for 48 h. We found that overexpression of circZKSaa remarkably repressed proliferation and induced apoptosis in HCC cell lines after sorafenib treatment (Fig. 3G). To further demonstrate the important role of polypeptide circZKSaa in the tumor suppressor effect, we constructed circZKSaa-mut Hep3B and HCC-LM3 cell lines. Later, we treated circZKSaa-mut Hep3B and HCC-LM3 cell lines with different concentrations of sorafenib, the results showed that ORF-mutated circZKSaa lost the ability to promote HCC apoptosis (Fig. S3A). This indicates that the polypeptide circZKSaa plays the unique role in inhibition of the tumorigenesis. Collectively, these findings demonstrated that circZKSaa may serve as a potential prognostic biomarker for HCC, and this circZKSaa peptide can enhance the anti-tumorigenesis of sorafenib in HCC cells.

### circZKSCaa inhibits the growth of HCC

**Fig. 4:**
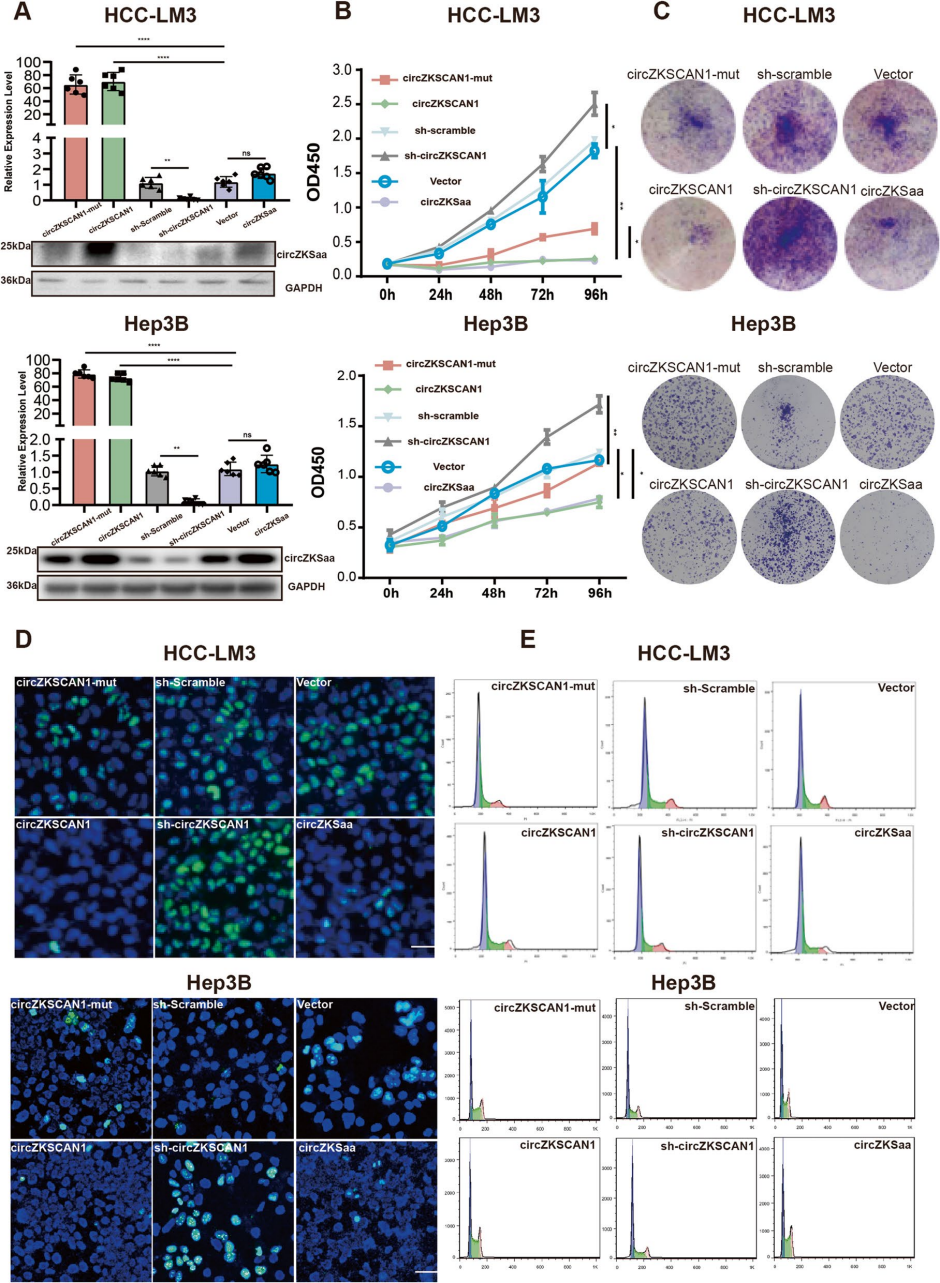
circZKSCaa inhibits tumor growth. **A** The mRNA and protein levels of circZKSaa and circZKSCAN1 in stable HCC-LM3 and Hep3B cells were demonstrated by real-time RT-qPCR and Western blot. Cell proliferation assay including **B** CCK8 analysis, **C** colony formation assays, **D** KI67 immunofluorescence assay, **E** Cell cycle progression was determined via flow cytometry assay for circZKSCAN1-mut, circZKSCAN1, sh-scramble, sh-circZKSCAN1, Vector and circZKSaa stable cell lines (HCC-LM3 and Hep3B). Quantitative data from three independent experiments are presented as mean ± SEM (error bars). Scale bars = 100 μm. T test was performed for significant analysis, significant differences are indicated with * for *p* < 0.05, ** for *p* < 0.01 and *** for *p* < 0.001

In order to explore the biological functions of circZKSaa in HCC cells, we constructed a series of stable cell lines such as linear circZKSaa-overexpressing cells, circZKSCAN1 overexpressing cells, mutant circZKSaa start codon cells (circZKSCAN1-mut), down-regulated cells (sh-circZKSCAN1), and also a sets of their respective control cells (sh-scramble and Vector), all stable cell lines were engineered based on HCC-LM3 and Hep3B cells (Fig. 4A). We detected the protein and RNA levels of circZKSCAN1 or circZKSaa by RT-qPCR and Western blot (Fig. 4A). The cell phenotype showed that the growth of HCC was inhibited in both overexpressed circZKSCAN1 and circZKSaa groups, rather than in circZKSCAN1-mut and vector groups (Fig. 4B and C). In the meantime, we noticed that the circZKSaa and circZKSCAN1 expression level was negatively correlated with the number of colony formation (Fig. 4C), the positive rate for KI67 was noticeable reduced by overexpression of circZKSaa and circZKSCAN1 (Fig. 4D). Furthermore, we found that HCC cells were significantly arrested at the percentage of G0/S phase while circZKSaa and circZKSCAN1 was overexpressed, which indicated that circZKSaa can act as a suppressor for the cell cycle (Fig. 4E, Fig. S3B).

### circZKSaa promotes tumor apoptosis

**Fig. 5:**
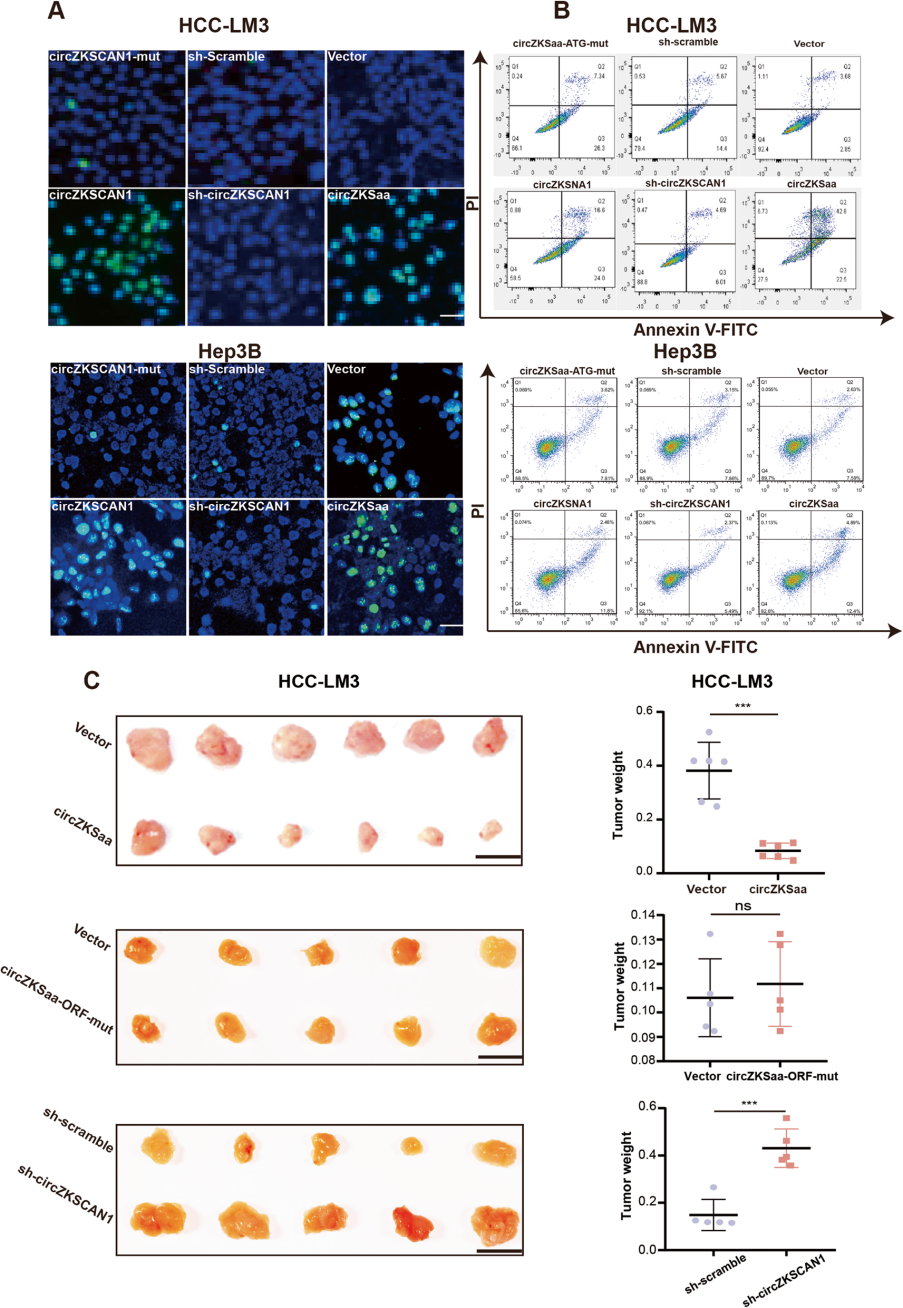
circZKSaa promotes tumor apoptosis. Cell apoptosis analyses contain **A** TUNEL staining and **B** Flow cytometry analysis for circZKSCAN1-mut, circZKSCAN1, sh-scramble, sh-circZKSCAN1, Vector and circZKSaa stable cell lines (HCC-LM3 and Hep3B). **C** Xenograft BALB/c nude mice (*n* = 5 or 6 each group) experiment analyses which including the images of tumors in each group and tumor volume to demonstrated the HCC cell growth in vivo*.* Quantitative data from three independent experiments are presented as mean ± SEM (error bars). Scale bars = 1 cm. T test was performed for significant analysis, significant differences are indicated with * for *p* < 0.05, ** for *p* < 0.01 and *** for *p* < 0.001

We further explore whether circZKSaa could lead to apoptosis in HCC cells. TUNEL staining demonstrated that overexpression of circZKSaa could promote apoptosis of HCC cells (Fig. 5A). In addition, we found that circZKSaa promoted early apoptosis in HCC cells by staining with Annexin V/PI (Fig. 5B).

To further explore the effects of circZKSaa in vivo, stable overexpressed circZKSaa and circZKSCAN1-mut HCC-LM3 cell lines were subcutaneously injected into the nude mice. We observed that tumor volume was smaller in circZKSaa overexpressed groups (Fig. 5C), whereas tumors in circZKSCAN1-mut were the same size as controls (Fig. 5C). Moreover, we observed larger tumor volumes in circZKSaa knockdown groups compared to the control groups (Fig. 5C). Collectively, the above analyses demonstrated that circZKSaa inhibits proliferation and promotes apoptosis in HCC cells.

### circZKSaa prevents AKT activation through mTOR

**Fig. 6:**
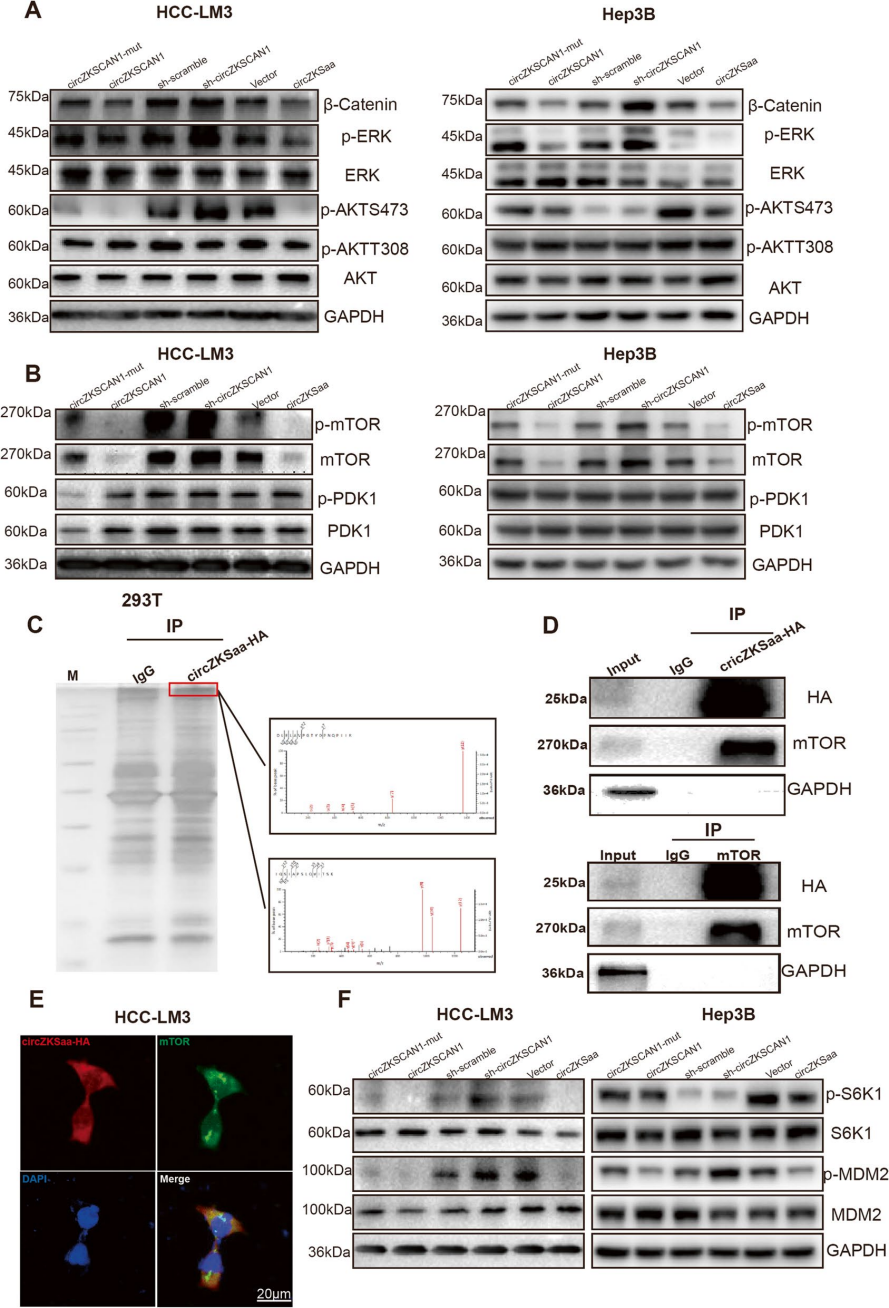
circZKSaa prevents AKT activation through mTOR. HCC-LM3 and Hep3B cells were transfected corresponding vectors (circZKSCAN1-mut\circZKSCAN1\sh-scramble\sh-circZKSCAN1\Vector\circZKSaa) to detect the proliferation signal pathway. **A** The expression level of β-catenin, ERK, phosphorylated ERK, AKT, T308 site phosphorylated-AKT and S473 site phosphorylated-AKT were determined via Western blot, and GAPDH expression level served to indicate the amount of loading proteins. **B** The protein expression level of PDK1, phosphorylated PDK1, mTOR and phosphorylated mTOR were determined via Western blot, and GAPDH expression level served to indicate the amount of loading proteins. **C** After Co-IP by HA antibody and separation by SDS-PAGE, the protein sample which stained by silver staining was analyzed by mass spectrometry. **D** Co-IP analyses by HA and mTOR antibody determined the interaction between circZKSaa and mTOR. **E** Immunofluorescence staining of mTOR and circZKSaa-HA in HCC-LM3 cells, Scale bars = 20 μm. **F** Western blot assay were used to detect MDM2, phosphorylated-MDM2, S6K1 and phosphorylated-S6K1 in both HCC-LM3 and Hep3B cells transfected with vectors (circZKSCAN1-mut\circZKSCAN1\sh-scramble\sh-circZKSCAN1\Vector\circZKSaa), GAPDH expression level indicated the amount of loading proteins. Quantitative data from three independent experiments are presented as mean ± SEM (error bars). T test was performed for significant analysis, significant differences are indicated with * for* p* < 0.05, ** for *p* < 0.01 and *** for *p* < 0.001

To further investigate the molecular mechanism of circZKSaa on inhibiting HCC, we checked the critical parameters involved in the tumorigenesis of HCC (Fig. 6A). We found several proliferation markers were changed, such as Phospho-ERK1/2 (Thr202/Tyr204), β-catenin and p-AKT-ser473 (Fig. 6A). Especially, p-AKT-ser473 was dramatically down-regulated, while no obvious change of p-AKT-thr308 was observed (Fig. 6A).

It was well known that AKT activation needs two steps: activated PDK1 phosphorylated at thr-308 site of AKT, and mTOR or Rictor-mTOR complexes continually phosphorylated the AKT ser-473 site in order to fully activate AKT [22, 23]. Western blot indicated that mTOR protein expression level was negatively associated with circZKSaa, while no significant changes were found in the expression of PDK1 (Fig. 6B). To examined whether mTOR or PDK1 has potential to interact with circZKSaa, we first carried the mass spectrometry analysis. We noticed that mTOR was one of interacting proteins in the HA-circZKSaa Co-IP complex (Fig. 6C). Co-IP results revealed that circZKSaa was detectable in mTOR antibody-mediated precipitate (Fig. 6D). Immunofluorescence staining in HCC-LM3 cells supported the co-localization of circZKSaa with mTOR (Fig. 6E). Moreover, we found circZKSaa overexpression inactivated phosphorylation of S6K1 and MDM2, confirmed that circZKSaa inhibited the downstream pathways of AKT and mTOR, respectively (Fig. 6F). These results demonstrated that circZKSaa acts as an HCC tumor suppressor via interacting with mTOR in vivo.

### circZKSCaa degrades mTOR by interacting with FBXW7

**Fig. 7:**
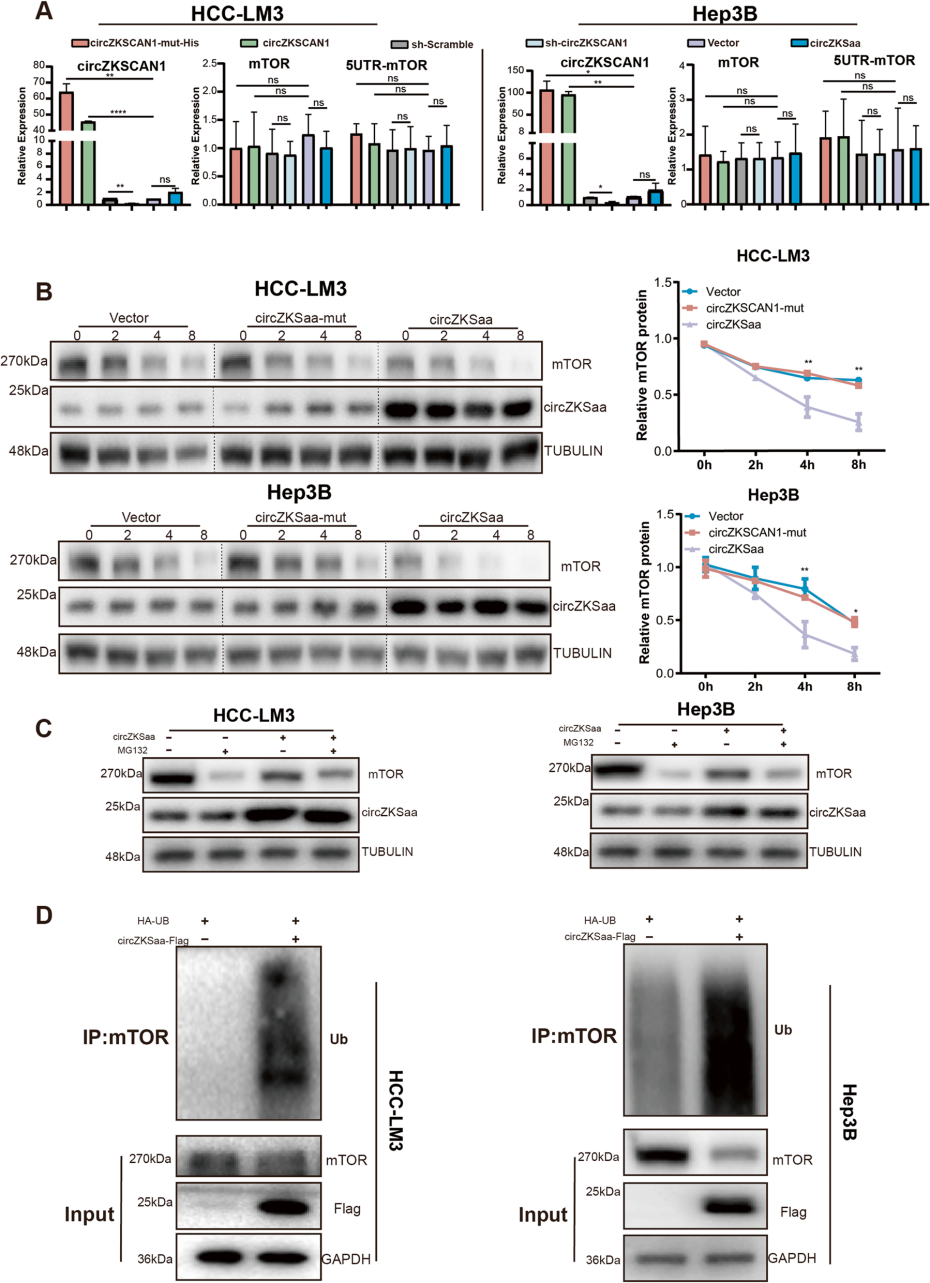
circZKSCaa promotes ubiquitination of mTOR. **A** RT-qPCR demonstrated mTOR and 5’UTR-mTOR expression level in circZKSCAN1-mut\circZKSCAN1\sh-scramble\sh-circZKSCAN1\Vector\circZKSaa transfected cells (HCC-LM3 and Hep3B). **B** Western blot detected half-life of mTOR by adding CHX for different times. The amount of mTOR protein level was calculated by ImageJ. **C** Western blot analyses mTOR protein degradation by MG-132 treatment. **D** Before treated with MG132, HCC-LM3 and Hep3B cells were transfected with HA-ubiquitin and circZKSaa-Flag. Whole cell lysates were IP with mTOR antibody followed by detection with ubiquitin antibody. GAPDH, Flag and mTOR serve as amount of input proteins. Quantitative data from three independent experiments are presented as mean ± SEM (error bars). T test was performed for significant analysis, significant differences are indicated with * for *p* < 0.05, ** for *p* < 0.01 and *** for *p* < 0.001

In order to figure out the molecular mechanism of circZKSaa in inhibiting mTOR, we performed a serial of assays. First of all, we found that the overexpression of circZKSaa does not significantly change the mRNA expression level of mTOR (Fig. 7A). Secondly, we performed a pulse chase experiment with cycloheximide (CHX) to inhibit new protein synthesis in HCC cells. The results showed remarkable degeneration of mTOR in circZKSaa group in comparison with vector and circZKSaa-ORF-mut groups (Fig. 7B). With a treatment of MG132 (the proteasome inhibitor), we found that the expression level of mTOR was restored significantly in circZKSaa overexpressed cell lines (Fig. 7C). Moreover, the ubiquitin-mediated proteasomal degradation of mTOR was enhanced when overexpressing circZKSaa in both cell lines (Fig. 7D).

**Fig. 8:**
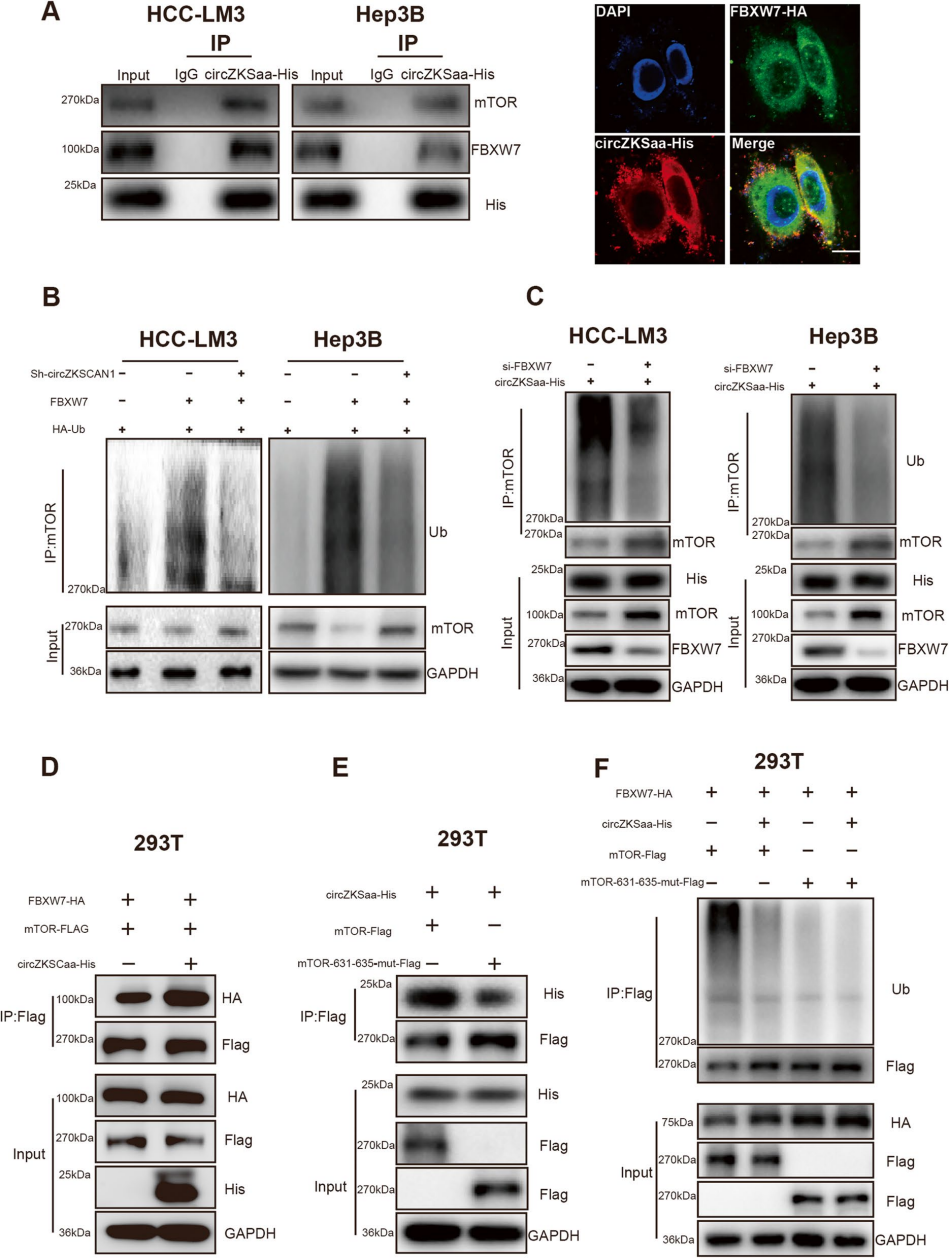
circZKSCaa degrades mTOR by interacting with FBXW7. **A** Co-IP assay by His antibody measured the interaction between mTOR, circZKSaa-His and FBXW7 in HCC-LM3 and Hep3B cells; Immunofluorescence staining of FBXW7-HA and circZKSaa-His in HCC-LM3 cells, Scale bars = 200 μm. **B** Western blot analyses mTOR ubiquitination level in FBXW7-overexpresion cells with or without down-regulated circZKSCAN1, both cells were pre-incubated with MG-132. **C** Western blot analyses mTOR ubiquitination level in circZKSaa-His overexpresion cells with or without down-regulated FBXW7, both cells were pre-incubated with MG-132. **D** FBXW7-HA, mTOR-Flag, circZKSaa-His vector plasmid was transfected into 293 T cells as indicated. Immunoprecipitation was performed with anti-Flag protein A/G magnetic beads and proteins from the immunoprecipitatte was subjected to Western blot for evaluation of FBXW7-HA and mTOR-Flag. **E** mTOR-Flag, mTOR-631/635-mut-Flag, circZKSaa-His vector plasmid was transfected into 293 T cells as indicated. Immunoprecipitation was performed with anti-Flag protein A/G magnetic beads and proteins from the immunoprecipitatte was subjected to Western blot for evaluation of circZKSaa-His and mTOR-Flag. **F** FBXW7-HA, mTOR-Flag, mTOR-631/635-mut-Flag, circZKSaa-His vector plasmid was transfected into 293 T cells as indicated. Immunoprecipitation was performed with anti-Flag protein A/G magnetic beads and proteins from the immunoprecipitatte was subjected to Western blot for evaluation of Ub and mTOR-Flag

It is well known that FBXW7 recognizes their substrates, such as cyclin E and mTOR, through the presence of a conserved CDC4 phosphorylation degrader (CPD) motif [24–27], which requires substrates to be phosphorylated at specific residues in order to be ubiquitinated and targeted for proteasomal degradation. We speculate that circZKSaa may affect the protein level of mTOR through FBXW7 protein. By Co-IP assay, we showed that circZKSaa and FBXW7 could interact with each other (Fig. 8A). Immunofluorescence staining exhibited that circZKSaa co-localized with FBXW7 in HCC cells (Fig. 8A). FBXW7-mediated mTOR ubiquitination was partially alleviated by knockdown of circZKSaa (Fig. 8B). Furthermore, we knocked down FBXW7 in circZKSaa overexpressed HCC cell lines, and the results showed that knockdown of FBXW7 resulted in the decreased ubiquitination of mTOR (Fig. 8C). These results suggest that circZKSaa may affect the ubiquitination level of mTOR protein through FBXW7 protein. To investigate whether circZKSaa affects the activity of other substrates of FBXW7, we detected the protein level of cyclin E in circZKSaa overexpressed HCC cell lines, and the results showed that overexpression of circZKSaa did not affect the protein level of cyclin E (Fig. S3D). This indicates that the interaction between circZKSaa and FBXW7 protein may be specifically related to mTOR. This may be due to the specific function of the polypeptide circZKSaa in HCC. Then, we speculate that circZKSaa may affect the stability of mTOR by affecting the interaction between FBXW7 and mTOR protein. The results showed that the binding of FBXW7 to mTOR was increased when circZKSaa was overexpressed in 293 T cells (Fig. 8D).

Next, we wanted to explore the detailed molecular mechanism of how circZKSaa promotes FBXW7-mediated mTOR degradation. The ubiquitination reaction of mTOR occurs only after the key sites 631 and 635 of mTOR recognized by FBXW7 [24, 27]. Since the 631 and 635 sites of mTOR are the most critical regions recognized by FBXW7, we wanted to first explore whether circZKSaa binds to the 631 and 635 regions of mTOR to affect the binding between mTOR and FBXW7. Immunoprecipitation and immunofluorescence experiments demonstrated that mTOR could bind to circZKSaa (Fig. 6D and E, Fig. 8A). After mutations in the 631 and 635 regions of mTOR, circZKSaa cannot interact with mTOR (Fig. 8E). Next, we wanted to explore the effect of mutations in the 631 and 635 regions of mTOR on Fbw7-mediated mTOR degradation. Our results showed that overexpression of circZKSaa in HCC cells led to increased ubiquitination of mTOR (Fig. 8F). This ubiquitination level of mTOR decreased when the 631 and 635 regions of mTOR were mutated (Fig. 8F).

## Discussion

In this study, we proved that circZKSCAN1 encodes a novel peptide circZKSaa, which is dramatically decreased in HCC tissues. And circZKSaa could sensitize HCC cells to sorafenib treatment. Mechanically, we demonstrated that circZKSCAN1 almost has no function as microRNA sponge, but circZKSaa inhibited AKT by enhancing the ubiquitination of mTOR, which in turn inhibited proliferation and promoted apoptosis of HCC cells. Our findings provide a novel insight that circZKSaa has a great potential to be a novel therapeutic target and diagnostic marker for HCC.

circZKSCAN1 has been reported to be involved in the tumorigenesis and tumor progression. Bi et al. have reported that circZKSCAN1 upregulated p21 expression by sponging miR-1178-3p, which suppressed the aggressive biological behaviors in bladder cancer [13, 20]. Wang et al. have proved that circZKSCAN1/miR-330-5p/FAM83A feedback loop promoted the progress of non-small-cell lung cancer [13]. Yao et al. have found that circZKSCAN1 regulates a variety of cancer pathways to inhibit the proliferation and migration of HCC cells by RNA-seq analysis. However, the exact molecular mechanism of how circZKSCAN1 inhibit the HCC progression remains unclear [28]. Strikingly, Zhu et al. found that circZKSCAN1 was a potential stemness regulator of HCC, and suppressed the transcriptional activity of Wnt/β-catenin signal pathway through competitively binding to FMRP in HCC [13]. Although studies by Zhu et al. did not mention the microRNA sponging function of circZKSCAN1 in HCC, it opened up a new perspective of circZKSCAN1 on understanding of the tumorigenesis of HCC [25]. Recently, Li et al. have found that circZKSCAN1 performs the function as a sponge to interact with microRNA-873-5p in HCC by dual luciferase reporter assay [29]. However, by using the more precise approach (RIP experiment of AGO2 protein), we demonstrated that circZKSCAN1 is hardly to act as a sponge to interact with microRNA in HCC cells. Consistent with the above studies, we also found that circZKSCAN1 participated in the development of HCC cells (Fig. 4A-E). Intriguingly, we found that circZKSaa inhibits the HCC cells growth by promoting the degradation of mTOR. Combined with our results, circRNA may elicit many actions in the etiology and pathology of same diseases. To Clarify which factors and how these factors regulate different functions of circZKSCAN1, further studies are urgent and needed.

circZKSCAN1 consists of exons 2 and 3 of ZKSCAN1 (Zinc-finger protein with KRAB and SCAN domains 1) spliced together to form a covalently linked 668-nt circular RNA [28, 30]. ZKSCAN1 mRNA mainly regulated cellular metabolism, and its specific downstream targets are scarce studied [31–33]. Studies have shown that the expression level of circZKSCAN1 is significantly correlated with the disease process of HCC, but there is no significant difference in the expression level of ZKSCAN1 between HCC and adjacent tissues [12]. In our study, we found that the expression of circZKSaa is significantly reduced in cancer tissues, while the expression of ZKSCAN1 did not change obviously (Fig. S2D). Therefore, we believed that circZKSaa inhibited HCC deterioration may through other signal pathways. Moreover, the upstream mechanisms governing the equilibrium between ZKSCAN1, circZKSCAN1 and circZKSaa in HCC need to be further investigated.

Our results proved that the overexpression of circZKSaa lead to the inhibited malignantcy in HCC cells. RNA binding protein QKI5 was the upstream of circZKSCAN1 and served as alternative splcing factor to regulated thousands of circRNAs formation so as to affect circZKSCAN1 expression [34]. We demonstrated the binding site of *QKI-5 *to circZKSCAN1 by RIP experiments. Furthermore, we found that the expression of *QKI-5* was upregulated after sorafenib treatment, which may be responsible for the upregulation of circZSKSCAN1. Furthermore, we demonstrated that circZKSaa can inhibit PI3K/AKT/mTOR, a classically dysregulated pathway involved in HCC pathogenesis [35], by promoting mTOR ubiquitination. mTOR has always been considered as an effective cancer therapeutic target, and the mTOR signaling involves complicated feedback networks [36, 37]. Whereas how circZKSaa captures the abnormally mTOR and regulates the related pathways are still our concern, which need to be studied in the near future.

In our study, we showed that circZKSaa was significantly up-regulated upon sorafenib treatment, and sensitize HCC cells to sorafenib treatment through ubiquitinated mTOR. There are a variety of factors contributing to the development of sorafenib resistance in HCC, such as the process of methylation and hypoxia, autophagic cell death and autophagy [38]. Of particular, the AKT/mTOR is a crucial hub for multiple signal pathways [38–41]. Our research proved that circZKSaa can degraded mTOR and inhibited the activation of AKT, leading to the inhibition of the proliferation of HCC cells. However, we still lack sufficient evidence to prove the relationship between mTOR and HCC drug resistance. In the future study, we are going to induce drug-resistant cell lines in vitro, and test circZKSaa for sorafenib sensitivity. We are also going to induce sorafenib resistance conditions in mice which bearing HCC tumour xenografts, and then inject circZKSaa into mice to observe whether it improves the sensitivity of HCC tissue. The specific molecular process is also our next research direction.

We also demonstrated that circZKSaa could be secreted into the supernatant of cell culture medium. This suggests that the polypeptide has the potential to work as a biomarker in HCC patients. We made great efforts to collect the serum of HCC patient and detect the expression level of circZKSaa, but unfortunately, we did not detect a clear positive band from the sera of HCC patients. This might be because the expression level of circZKSaa in the sera of HCC patients is too low, which under the detection limit of Western bolt. To solve this problem, we also tried LC-MS detection by an isotope-labeled specific probe, but we couldn’t detect the circZKSaa-specific sequences by LC-MS neither. The reason for this failure may be due to the amino acid sequence of this polypeptide circZKSaa is only 13 aa different from that of the maternal ZKSCAN1, this limits the sensitivity and specificity of the probe. Fortunately, by searching and analyzing a large number of databases, we found that the RNA level of circZKSCAN1 is low in HCC serum (Fig. 3D). This suggests that circZKSCAN1 may be a useful biomarker for HCC patients. Since RNA-seq can amplify the detection signal of mRNA, although the expression level of circZKSCAN1 in the serum of HCC patients is very low, it can still be detected by RNA-seq. In order to increase the detection specificity and sensitivity for this peptide, we will make the antibody for ELISA assay in our future study. As this translatable circZKSaa elicits its action in a way of polypeptide rather than in the way of nucleotide in liver tissue, we proposed that is better than nucleotides circZKSCAN1 as a biomarker for the clinical usage. Of course, to further confirm this polypeptide circZKSaa as a biomarker, the clinical investigation with large-scale of HCC patients are warrant.

## Conclusions

In summary, our study exhibited that circZKSCAN1 encoded a novel peptide-circZKSaa, which was is upregulated by *QKI-5* after sorafenib treatment and then increased the sensitivity of HCC cells to sorafenib treatment. Overexpression of circZKSaa inhibited the proliferation of HCC cell lines by FBXW7-mediated degradation of mTOR. Taken together, circZKSaa may serve as a feasible potential biomarker and the theraputic target for HCC treatment.

## Supplementary Information

**Additional file 1.**

**Additional file 2.**

## Data Availability

The datasets used and analyzed during the current study are available from the corresponding author on reasonable request.

## Abbreviations

circRNA: circular RNA
HCC: Hepatocellular carcinoma
hsa_circ_0001727: circZKSCAN1
co-IP: coimmunoprecipitation
DAPI: 4′, 6-diamidino-2-phenylindole
GAPDH: Glyceraldehyde-3-phosphate dehydrogenase
gDNA: Genomic DNA
SEM: Standard error of mean
IRES: Ribosome entry site
ORF: Open reading frame
Rluc–Luc: Renilla luciferase-Luciferase
OE circZKSCAN1: circZKSCAN1 overexpressing plasmid
OE circZKSaa: Linearized circZKSaa overexpressing plasmid
sh-circZKSCAN1: Knock down circZKSCAN1 expression plasmid
circZKSCAN1-mut: Overexpressed circZKSCAN1 with mutated circZKSaa start codon
sh-scramble: Knock-down control plasmid
Vector: Control overexpressing plasmid
ZKSCAN1: Zinc-finger protein with KRAB and SCAN domains 1
FMRP: Fragile X mental retardation protein
mTOR: Mammalian target of rapamycin
ROC: Receiver operating characteristic
