# Supplementary material for: A novel polypeptide encoded by the circular RNA ZKSCAN1 suppresses HCC via degradation of mTOR
Source: Mol Cancer. 2023 Jan 23;22:16. doi: 10.1186/s12943-023-01719-9 (PMC9869513; doi:10.1186/s12943-023-01719-9)
Supplement: Supplementary file 1 — Additional file 1. [file 12943_2023_1719_MOESM1_ESM.pdf]

Supporting Figure Legend

Fig. S1

H) RT-PCR validation results of the circRNAs in blank (left) and RNaseR treated (right) HCC-LM3 cells. B) Relative expression level of the 4 circRNAs in normal hepatocyte lines (THL-2) and HCC cell lines by real-time qRT-PCR analysis. C) RT-PCR and schematic illustration showed hsa_circ_0001727, hsa_circ_0006886 and hsa_circ_0071106 expression upon RNaseR treatment in HCC-LM3 cells, and sequencing showed hsa_circ_0001727, hsa_circ_0006886 and hsa_circ_0071106 back-splice site. D) Relative Expression level of circZKSCAN1 in both HCC-LM3 and Hep3B cells with RNaseR treatment. E) IC50 assay detected HCC-LM3 and Hep3B cells with or without circZKSaa transfection after different sorafenib treatment. F) Analysis of apoptosis in HCC-LM3 and Hep3B with or without knockdown circZKSCAN1 transfection by flow cytometry assays. Quantitative data from three independent experiments are presented as mean±SEM (error bars). T test was performed for significant analysis, significant differences are indicated with * for $p<0.05$, ** for $p<0.01$ and *** for $p<0.001$.

Fig. S2

A) CircInteractome website showed the potentially binding RBPs with circZKSCAN1. B) RIP analysis of circZKSCAN1, linZKSCAN1 using antibodies against AGO2. The RIP enrichment of the AGO2-associated circRNAs (as indicated) was measured by real time qRT-PCR, and each value was normalized to the level of

input RNA used in RIP analysis. linZKSCAN1 serve as the negative control and circHIPK3 as the positive control. C) circRNADb website datasets showed IRES regions and the potentially peptide translated by circZKSCAN1. D) Western blot showed expression of ZKSCAN1 in HCC-LM3 cells which transfected with circZKSCAN1-mut, circZKSCAN1, sh-scramble and sh-circZKSCAN1, Vector and circZKSaa. E) The circZKSaa content was tested via Western blot in 17 pairs of HCC and normal tissues. β-actin was used to indicate the amount of loading proteins. Quantitative data from three independent experiments are presented as mean±SEM (error bars). T test was performed for significant analysis, significant differences are indicated with * for $p<0.05$, ** for $p<0.01$ and *** for $p<0.001$.

Fig. S3

A) Analysis of apoptosis in HCC-LM3 and Hep3B with or without knockdown circZKSCAN1-mut transfection by flow cytometry assays. B) Cell cycle assays were used to detect cell cycle arrest levels of circZKSCAN1-mut, circZKSCAN1, sh-scramble, sh-circZKSCAN1, Vector and circZKSaa stable cell lines (HCC-LM3 and Hep3B). C) Apoptosis flow cytometry analysis for circZKSCAN1-mut, circZKSCAN1, sh-scramble, sh-circZKSCAN1, Vector and circZKSaa stable cell lines (HCC-LM3 and Hep3B). D) The expression level of cyclin E were determined via Western blot, and TUBULIN expression level served to indicate the amount of loading proteins. Quantitative data from three independent experiments are presented as mean±SEM (error bars). T test was performed for significant analysis, significant differences are indicated with * for $p<0.05$, ** for $p<0.01$ and *** for $p<0.001$.

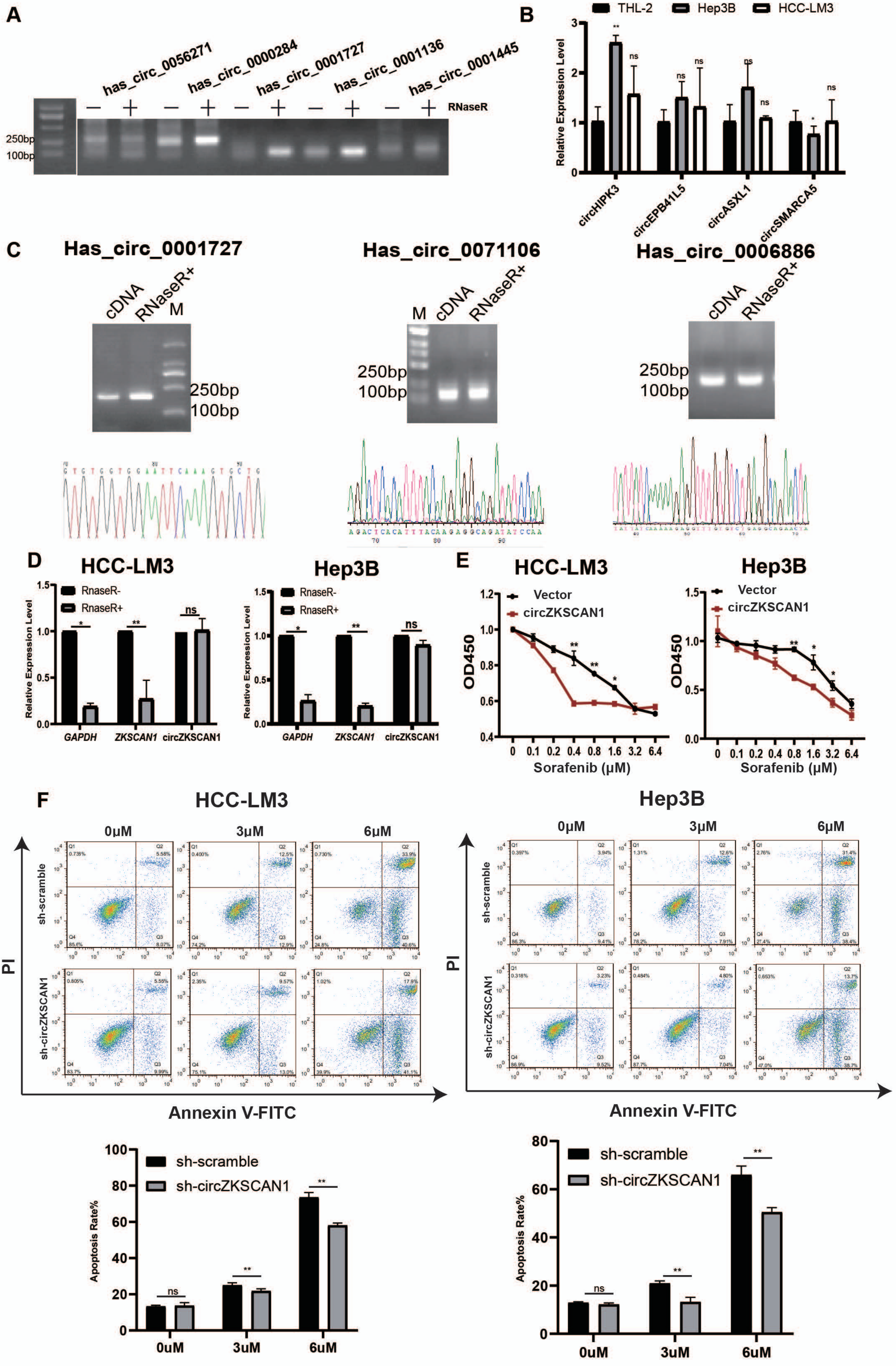

A
has_circ_0056271
has_circ_0000284
has_circ_0001727
has_circ_0001136
has_circ_0001445
RNaseR
250bp
100bp
B
THL-2
Hep3B
HCC-LM3
Relative Expression Level
circHIPK3
circEPB41L5
circASXL1
circSMARCA5
C
Has_circ_0001727
Has_circ_0071106
Has_circ_0006886
cDNA
RNaseR+
M
250bp
100bp
D
HCC-LM3
Hep3B
RnaseR-
RnaseR+
Relative Expression Level
GAPDH
ZKSCAN1
circZKSCAN1
E
HCC-LM3
Hep3B
Vector
circZKSCAN1
OD450
Sorafenib (μM)
F
HCC-LM3
Hep3B
0μM
3μM
6μM
sh-scramble
sh-circZKSCAN1
PI
Annexin V-FITC
sh-scramble
sh-circZKSCAN1
Apoptosis Rate%
0uM
3uM
6uM

A

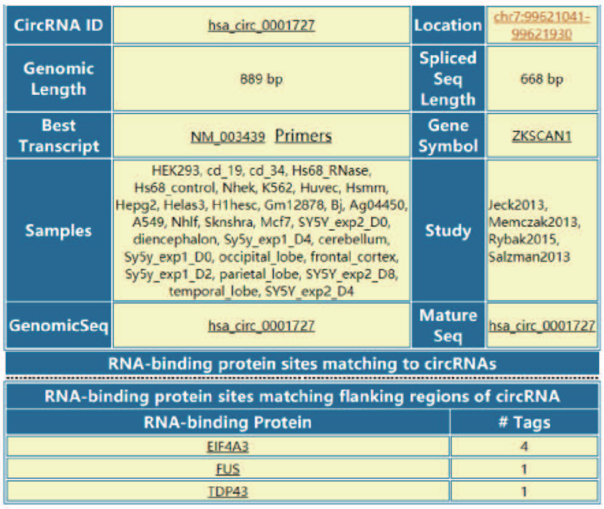

CircRNA ID	hsa_circ_0001727	Location	chr7:99621041-99621930
Genomic Length	889 bp	Spliced Seq Length	668 bp
Best Transcript	NM_003439 Primers	Gene Symbol	ZKSCAN1
Samples	HEK293, cd_19, cd_34, Hs68_RNase, Hs68_control, Nhek, K562, Huvec, Hsmm, Hepg2, Helas3, H1hesc, Gm12878, Bj, Ag04450, A549, Nhlf, Sknshra, Mcf7, SY5Y_exp2_D0, diencephalon, Sy5y_exp1_D4, cerebellum, Sy5y_exp1_D0, occipital_lobe, frontal_cortex, Sy5y_exp1_D2, parietal_lobe, SY5Y_exp2_D8, temporal_lobe, SY5Y_exp2_D4	Study	Jeck2013, Memczak2013, Rybak2015, Salzman2013
GenomicSeq	hsa_circ_0001727	Mature Seq	hsa_circ_0001727

RNA-binding protein sites matching to circRNAs

RNA-binding protein sites matching flanking regions of circRNA

RNA-binding Protein	# Tags
EIF4A3	4
FUS	1
TDP43	1

B

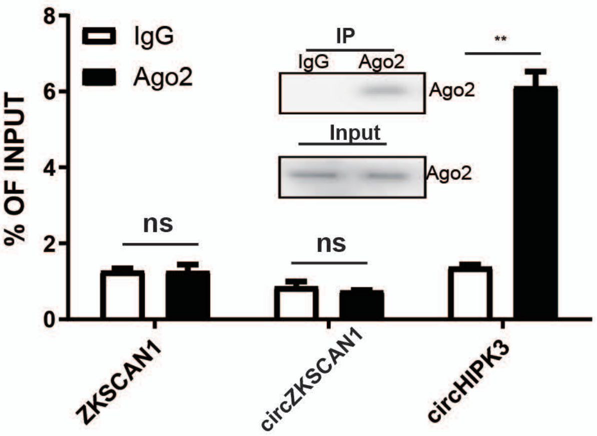

C

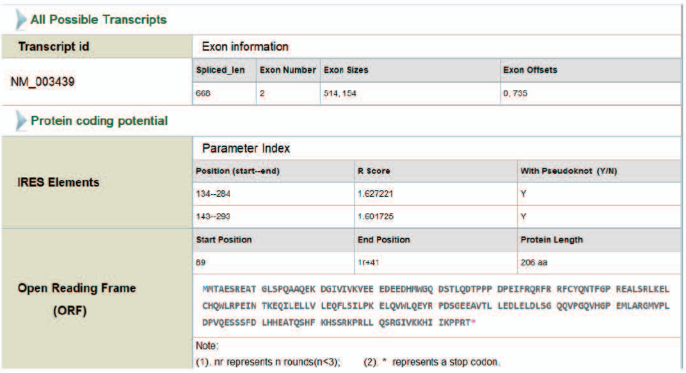

All Possible Transcripts

Transcript id	Exon information			
	Spliced_len	Exon Number	Exon Sizes	Exon Offsets
NM_003439	668	2	514, 154	0, 735

Protein coding potential

	Parameter Index		
IRES Elements	Position (start--end)	R Score	With Pseudoknot (Y/N)
	134--284	1.627221	Y
	143--293	1.601725	Y
Open Reading Frame (ORF)	Start Position	End Position	Protein Length
	89	1r+41	206 aa
	MMTAESREAT GLSPQAAQEK DGIVIVKVEE EDEEDHMWGQ DSTLQDTPPP DPEIFRQRFR RFCYQNTFGP REALSRLKEL CHQWLRPEIN TKEQILELLV LEQFLSILPK ELQVWLQEYR PDSGEEAVTL LEDLELDLSG QQVPGQVHGP EMLARGMVPL DPVQESSSFD LHHEATQSHF KHSSRKPRLL QSRGIVKKHI IKPPRT*		
	Note: (1). nr represents n rounds(n<3); (2). * represents a stop codon.		

D

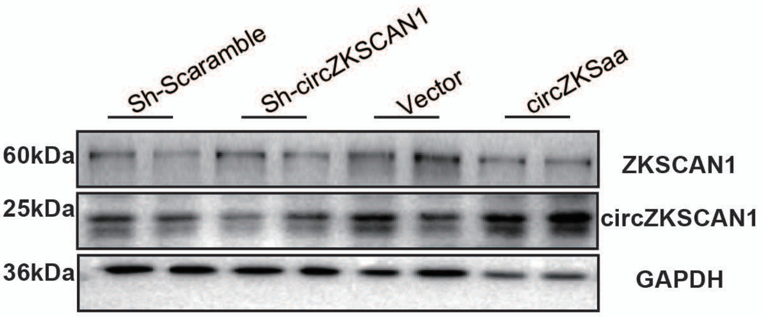

E

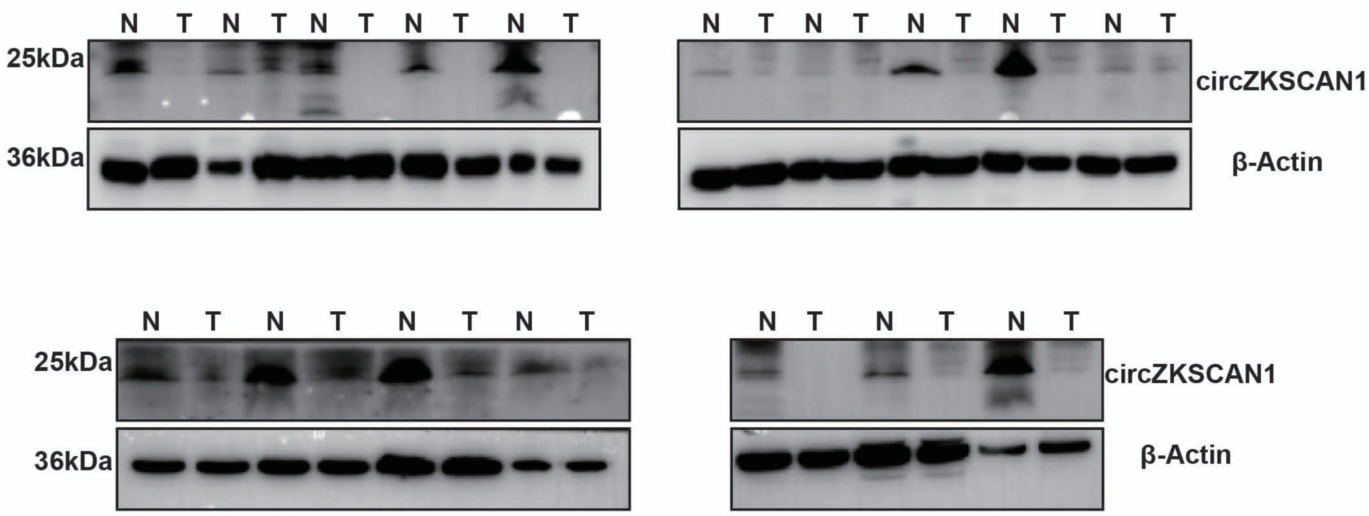

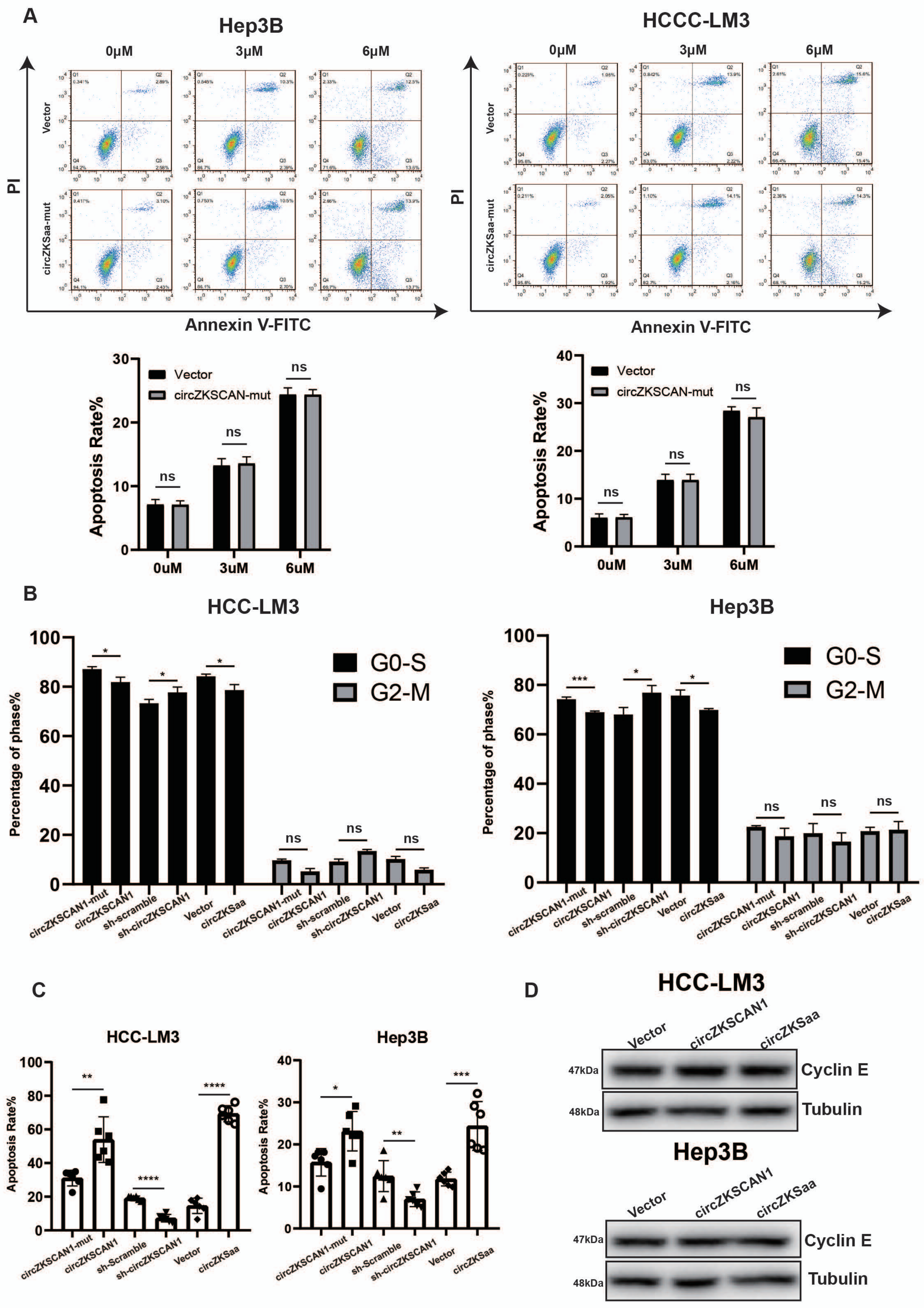
A
Hep3B
0μM
3μM
6μM
Vector
circZKSaa-mut
PI
Annexin V-FITC
HCCC-LM3
0μM
3μM
6μM
Vector
circZKSaa-mut
PI
Annexin V-FITC
Apoptosis Rate%
Vector
circZKSCAN-mut
ns
0uM
3uM
6uM
Apoptosis Rate%
Vector
circZKSCAN-mut
ns
0uM
3uM
6uM
B
HCC-LM3
Percentage of phase%
G0-S
G2-M
circZKSCAN1-mut
circZKSCAN1
sh-scramble
sh-circZKSCAN1
Vector
circZKSaa
Hep3B
Percentage of phase%
G0-S
G2-M
circZKSCAN1-mut
circZKSCAN1
sh-scramble
sh-circZKSCAN1
Vector
circZKSaa
C
HCC-LM3
Apoptosis Rate%
circZKSCAN1-mut
circZKSCAN1
sh-Scramble
sh-circZKSCAN1
Vector
circZKSaa
Hep3B
Apoptosis Rate%
circZKSCAN1-mut
circZKSCAN1
sh-Scramble
sh-circZKSCAN1
Vector
circZKSaa
D
HCC-LM3
Vector
circZKSCAN1
circZKSaa
47kDa
Cyclin E
48kDa
Tubulin
Hep3B
Vector
circZKSCAN1
circZKSaa
47kDa
Cyclin E
48kDa
Tubulin
